# The Erie Railway

**Published:** 1877-09

**Authors:** 


A very large proportion of the travelers over our great railway lines, particularly those which extend to popular resorts, such as Niagara Falls, the Watkins Glen, and the other numerous attractive objects within a day's journey of New York, are pleasure travelers seeking rest and recreation for themselves and their families. To them the most attractive route, the one which presents the finest scenery, if at the same time safe, comfortable and rapid, is the route to be selected. To the man of business who selects his routes with reference to their comfort and speed, it is certainly no objection to any road that it conducts him through grand and attractive scenery.

No one destined to the thousands of attractive spots in the interior of the State, to the lake region of Central New York, with its beautiful scenery,—glens and waterfalls, its springs and pleasant rural retreats, would be likely to find his way to them by any other than the Erie Railway, if he were well informed as to its superior attractions. When the traveler has passed the fertile and pleasant plains of New Jersey, over which the first few miles of his journey lies, he enters a wilder and more attractive region abounding in the grand and the picturesque. Few railway journeys in the world present so great a variety of attractive scenery as the Erie route from Ramapo along the Delaware, to the Susquehanna, and thence to the Chemung. Near its line are many spots full of interest to the tourist, and especially attractive to the seeker after health. Summer hotels, fishing grounds, health giving springs, and natural scenery of every variety of attractiveness are found from one end of the road to the other.

Of its great rival, the New York Central, little can be said as a route for those who desire to combine the attractiveness of fine scenery with luxury in travel. The Hudson river should never be viewed from the railway. Beyond the Hudson the Central abounds in pleasant views over a rich and well cultivated country, and passes the outskirts of thrifty and pleasant towns, but it presents to the traveler no object to attract the attention of those who delight in the grand or the beautiful.

